# Long-term Exposure to Traffic-related Air Pollution and Type 2 Diabetes Prevalence in a Cross-sectional Screening-study in the Netherlands

**DOI:** 10.1186/1476-069X-10-76

**Published:** 2011-09-05

**Authors:** Marieke BA Dijkema, Sanne F Mallant, Ulrike Gehring, Katja van den Hurk, Marjan Alssema, Rob T van Strien, Paul H Fischer, Giel Nijpels, Coen DA Stehouwer, Gerard Hoek, Jacqueline M Dekker, Bert Brunekreef

**Affiliations:** 1 Department of Environmental Health, Public Health Service Amsterdam, Amsterdam, the Netherlands; 2 Institute for Risk Assessment Sciences, Utrecht University, Utrecht, the Netherlands; 3 EMGO Institute for Health and Care Research, VU University Medical Center, Amsterdam, the Netherlands; 4 Centre for Environmental Health Research, National Institute for Public Health and the Environment (RIVM), Bilthoven, the Netherlands; 5 Department of Internal Medicine and Cardiovascular Research Institute Maastricht, Maastricht University Medical Centre, Maastricht, the Netherlands; 6 Department of Epidemiology and Biostatistics, VU University Medical Center, Amsterdam, the Netherlands; 7 Julius Center for Health Sciences and Primary Care, University Medical Center Utrecht, Utrecht, the Netherlands

**Keywords:** 50-75 yrs, general population, long term, the Netherlands, traffic related air pollution, type 2 diabetes

## Abstract

**Background:**

Air pollution may promote type 2 diabetes by increasing adipose inflammation and insulin resistance. This study examined the relation between long-term exposure to traffic-related air pollution and type 2 diabetes prevalence among 50- to 75-year-old subjects living in Westfriesland, the Netherlands.

**Methods:**

Participants were recruited in a cross-sectional diabetes screening-study conducted between 1998 and 2000. Exposure to traffic-related air pollution was characterized at the participants' home-address. Indicators of exposure were land use regression modeled nitrogen dioxide (NO_2_) concentration, distance to the nearest main road, traffic flow at the nearest main road and traffic in a 250 m circular buffer. Crude and age-, gender- and neighborhood income adjusted associations were examined by logistic regression.

**Results:**

8,018 participants were included, of whom 619 (8%) subjects had type 2 diabetes. Smoothed plots of exposure versus type 2 diabetes supported some association with traffic in a 250 m buffer (the highest three quartiles compared to the lowest also showed increased prevalence, though non-significant and not increasing with increasing quartile), but not with the other exposure metrics. Modeled NO_2_-concentration, distance to the nearest main road and traffic flow at the nearest main road were not associated with diabetes. Exposure-response relations seemed somewhat more pronounced for women than for men (non-significant).

**Conclusions:**

We did not find consistent associations between type 2 diabetes prevalence and exposure to traffic-related air pollution, though there were some indications for a relation with traffic in a 250 m buffer.

## Background

Many different factors are involved in the development of type 2 diabetes. Genetic predisposition, excess caloric intake and reduced physical activity are established and well-known determinants [1]. It has recently been hypothesized that long-term exposure to traffic-related air pollution might be an environmental risk factor for type 2 diabetes [2-5].

Epidemiological studies have demonstrated that long-term exposure to traffic-related air pollution is associated with an increased risk for cardiopulmonary morbidity and mortality [6,7]. An hypothesis for the biological mechanism underlying these associations is that traffic-related air pollution triggers systemic oxidative stress and inflammation in for instance endothelial cells and macrophages [7,8]. These biological mechanisms are known to be involved in the development of insulin resistance seen in type 2 diabetes [9,10]. Consequently, it seems plausible that exposure to traffic-related air pollution could also be a risk factor for type 2 diabetes, like environmental tobacco smoke is [11]. At present, there is little data supporting this hypothesis. Recently, Sun et al. [4] demonstrated increased adiposity inflammation and whole-body insulin resistance in mice exposed to particulate matter air pollution. A study by Kramer et al. [3] further supported the plausibility of oxidative stress and inflammation as a biological mechanism for the relation between air pollution and type 2 diabetes, by showing that women with high C3c blood levels (a marker for subclinical inflammation) were more susceptible for particulate matter related excess risk of diabetes than were women with low C3c levels. That prospective study furthermore found a relation between traffic-related particulate matter and incident type 2 diabetes among elderly women in Germany [3]. Another epidemiological study, by Brook et al. [2], found an association between modeled NO_2 _exposure and type 2 diabetes prevalence among female patients, but not among male patients, of two respiratory health clinics in Canada. In addition, a recent American study found an association with distance to road among women, while no strong evidence of an association with particulate matter exposure was observed [5].

The objective of the present study was to examine the relation between long-term exposure to traffic-related air pollution at the home-address and type 2 diabetes prevalence among subjects aged 50 to 75 years, living in a semi-rural region of the Netherlands.

## Methods

### Study area and study population

**Figure 1:**
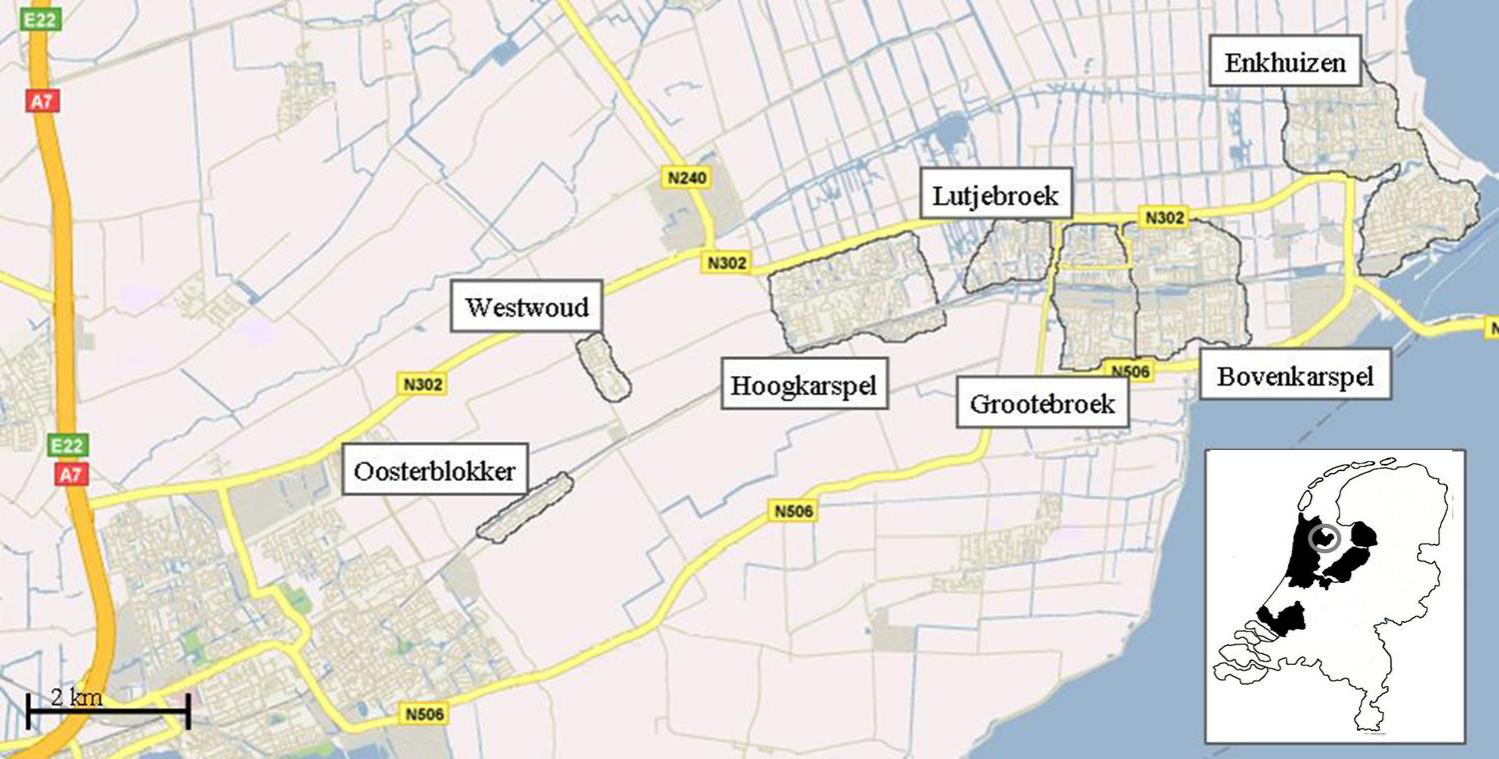
**Study area and overview of specific location in the Netherlands**. The study area consisted of three municipalities. Shown are the seven towns or villages within these municipalities, the highways (provincial roads) adjacent to the area and the nearest freeway, which is located to the west of the study area. The circle within the map of the Netherlands indicates were the study area is situated, the area marked in black is the area the NO_2_-model was developed for.

The study was performed among residents of the semi-rural area of Westfriesland in the North-West of the Netherlands (Figure 1). The study area comprised three municipalities, consisting of seven towns and villages (Enkhuizen, Bovenkarspel, Grootebroek, Lutjebroek, Hoogkarspel, Westwoud and Oosterblokker). A large proportion of the estimated surface of 56 km^2 ^is used for agricultural activities, typically horticulture of tulips and cauliflower. Residents often commute to work in the area of Amsterdam, around 60 km away. No freeways are present in the study area. Two highways, known as provincial roads in the Netherlands, with a traffic flow of approximately 15,000 to 25,000 vehicles/24 hrs, outline the North and South borders of the study area and are connected with the nearest freeway, located approximately 4 km to the west of the study area.

The study population has been described in more detail elsewhere [12]. In brief, between 1998 and 2000, all 50- to 75-year-old residents of the study area were invited to participate in the Hoorn Screening Study for type 2 diabetes. A total of 11,679 inhabitants received an invitation letter and the Symptom Risk Questionnaire, a screening instrument for undetected type 2 diabetes, which contained nine questions about age, gender, body length, body weight, family history of diabetes and health related problems like pain when walking or frequent thirstiness [13]. BMI was derived of data on body length and -weight.

Of all responding participants (N = 8,153), 417 (5%) reported previously doctor diagnosed diabetes. Participants with previously diagnosed diabetes were not required to complete the Symptom Risk Questionnaire and were not screened further. For the remaining 7,736 participants, risk-scores were calculated from the questionnaire. Participants with scores indicating a high risk profile for undetected type 2 diabetes (n = 3,301) were asked to engage in further testing based on the 1999 World Health Organization guidelines for diagnosis of type 2 diabetes [14]. Further testing comprised fasting capillary glucose measurements. Depending on the outcomes of these capillary measurements, a venous fasting plasma glucose sample was taken, followed by either an oral glucose tolerance test or a second fasting plasma glucose measurement. The screening resulted in the diagnosis of 217 new cases of type 2 diabetes. Consequently, the Hoorn Screening Study population included 634 (8%) participants with type 2 diabetes.

The Dutch Central Bureau for Statistics provided additional population data on average monthly income of all residents in 2004 at a six-position postcode area level, which typically comprises about 20 dwellings.

### Exposure

Exposure to traffic-related air pollution was characterized at each participant's residential address at time of recruitment. All addresses were geocoded by means of the national GIS (Geographical Information System) database ACN [15], which contains coordinates for all home addresses in the Netherlands. Exposure to traffic-related air pollution was defined by four different variables that have been demonstrated to be valid indicators of exposure [16-19]: modeled NO_2_-concentration, distance to the nearest main road, traffic flow at the nearest main road and traffic within a 250 m circular buffer. NO_2 _is considered an indicator of the complex mix of various gaseous and particulate components originating from both traffic combustion and wear of road and vehicles.

NO_2_-concentrations at the home address were estimated by means of a land use regression model for the West of the Netherlands (Figure 1) that has been described elsewhere [20]. In brief, during one week in all four seasons of 2007, NO_2_-measurements were performed using passive samplers at a total of 60 urban traffic dominated-, urban background- and rural background sites distributed over a large area (6,000 km^2^) in the West of the Netherlands, of which the current study area is part of. Traffic flow data were provided by all national, provincial and municipal authorities in the study area and were linked to a digital map of all roads in the Netherlands (NWB), using GIS. Other land use data were obtained from a European land use database (CORINE). Supervised forward selection was used to construct the land use regression model. The predictors in the final model were: background NO_2_-concentration, traffic volume at the nearest road, distance to the nearest main road and residential land use in a 5 km circular buffer. The cross-validation, adjusted, model R^2 ^was 82% [20].

Furthermore, for each participants' residential address, other exposure indicators were derived from the traffic data described above using GIS: distance to the nearest main road (defined as a road with at least 5,000 vehicles/24 hrs), traffic flow at the nearest main road (number of vehicles/24 hrs), and total traffic per 24 hours on all roads within a 250 m circular buffer around the address. All GIS calculations were conducted using ArcInfo (ESRI, Redlands, CA).

### Statistical analyses

Participants with missing values on exposure variables and the covariates age, gender and income were excluded from all analyses. We used penalized regression splines as implemented by Wood [21] in R (GAM procedure, mgcv-package of R version 2.8.0, R foundation for Statistical Computing, Vienna, Austria) to explore the functional relation between type 2 diabetes prevalence and the exposure variables. Since associations with type 2 diabetes seemed to be nonlinear, all exposure variables were analyzed in quartiles. As this approach may have resulted in arbitrary intervals, which were sometimes quite narrow, smooth plots of the association between exposure and type 2 diabetes resulting from the GAM procedure were also presented for reference.

Logistic regression analysis was used to examine associations between type 2 diabetes prevalence and the different exposure variables. For each exposure variable, the quartile with the lowest level of exposure was chosen as the reference category. Analyses were performed with and without adjusting for a priori selected covariates age (continuous), gender, and average monthly income (continuous) as an indicator of neighborhood socio-economic status. Individually available covariates (gender, age and BMI) were also tested for effect modification. Stratified analyses were done by gender. Nationality was not adjusted for, as 99% of the population was Dutch. Since participants who reported previously diagnosed diabetes (n = 417) were not required to complete the Symptom Risk Questionnaire, data on BMI was missing for 98 of these respondents. To be able to include all patients in the main analyses, we decided not to adjust for BMI in the main analyses, but to perform a sensitivity analysis to explore the potential confounding effect of BMI. In the sensitivity analysis we compared the results of covariate-adjusted (all previously mentioned covariates with and without additional adjustment for BMI) logistic regression analyses for the subgroup of participants with non-missing information on BMI. Additional sensitivity analysis was performed for type of diagnosis (self-reported previously doctor diagnosed and screening diagnosed), excluding participants with type 2 diabetes from the other diagnosis group. For all exposure variables, odds ratios (OR) and 95% confidence intervals (95%-CI) are presented. All analyses (besides the GAM analyses) were done with SAS 9.2 (SAS Institute Inc., Cary, NC, USA).

## Results

**Figure 2:**
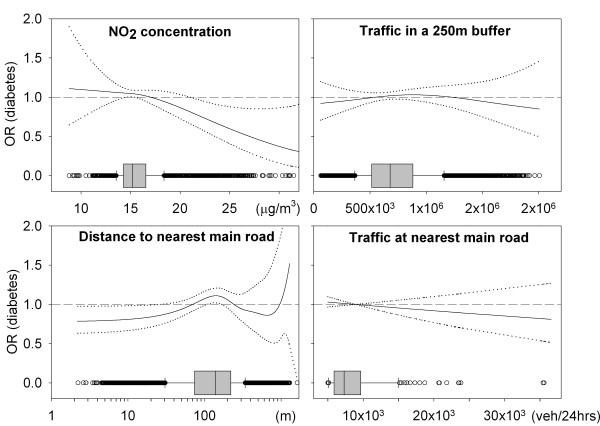
**Smooth adjusted associations (OR and 95%-CI) between exposure variables and type 2 diabetes prevalence**. Box plots on the x-axis present distribution of exposure variables.

Participants living outside the study area (n = 2), participants for whom geocoding of the home-address was not possible (due to a PO Box, boat or mail address, n = 11) and participants with missing data on the covariates gender, age and income (average monthly income, n = 118) were excluded from the study. This resulted in a study population of 8,018 participants, including 619 (8%) participants with type 2 diabetes, 406 previously diagnosed and 213 diagnosed in the Hoorn Screening Study. Forty-nine percent of the total population was male (Table 1) and median age of the total population was 58 years. The Box plots of the distribution of the exposure variables are presented in Figure 2. More detailed information about the distribution of the exposure variables and distributions for the participants with and without type 2 diabetes separately are presented in Additional File 1 Table s1. Additional File 1 Table s1 also shows the distribution of the predictors of the NO_2 _model. For one address the distance to the nearest busy road was outside the range of the distances for the monitoring sites based on which the model was developed (further away); all other predictors were within range of the original database [20]. Correlation between modeled NO_2_-concentration and distance to the nearest main road was high (Spearman's r: -0.88). Distance to the nearest main road and traffic in a 250 m buffer were also correlated (0.63), as were modeled NO_2_-concentration and traffic in a 250 m buffer (0.51). Traffic at the nearest main road was not correlated to the other exposure variables (r<0.2).

**Table 1:** Characteristics of the total population and of participants with and without type 2 diabetes.

Characteristic	Total population	Type 2 Diabetes (Total)	Screening diagnosed Type 2 Diabetes	No Type 2 Diabetes
	(N = 8018)	(N = 619)	(n = 213)	(N = 7399)

Gender (male)	3,949 (49%)	330 (53%)	111 (52%)	3,619 (49%)

Age (years)				

50-55	2,753 (34%)	96 (16%)	28 (13%)	2,657 (36%)

55-60	1,795 (22%)	110 (18%)	38 (18%)	1,685 (23%)

60-65	1,446 (18%)	122 (20%)	45 (21%)	1,324 (18%)

≥ 65	2,024 (25%)	291 (47%)	102 (48%)	1,733 (24%)

BMI (kg·m^-2^)				

< 18.5	51 (1%)	3 (1%)	1 (1%)	48 (1%)

18.5-25.0	3,632 (45%)	130 (21%)	34 (16%)	3502 (47%)

25.0-30.0	3,344 (42%)	243 (39%)	108 (51%)	3101 (42%)

≥ 30.0	893 (11%)	145 (23%)	70 (33%)	748 (10%)

missing	98 (1%)	98 (16%)	-	-

Average monthly income (€)	1,903 (417)	1,804 (407)	1,831 (464)	1,912 (417)

Total subjects with diabetes	619 (8%)	619 (100%)	213 (100%)	-

Subjects with pre-diagnosed diabetes	406 (5%)	406 (66%)	-	-

Data are number (%) or mean (sd).

Crude and adjusted associations between type 2 diabetes prevalence and the four indicators of exposure are shown in Additional File 1 Figure s1 (crude smooth plots), Figure 2 (gender, age and neighborhood income adjusted smooth plots) and Table 2 (exposure quartiles, crude and adjusted). Both smoothing splines and analyses by exposure quartiles first show a slight increase in prevalence of diabetes with increasing modeled NO_2_-concentration; then, when roughly modeled NO_2_-concentrations exceeded the 75-percentile, the prevalence decreased and fell below the prevalence at the lowest modeled NO_2_-concentrations. Overall, association between diabetes and modeled NO_2_-concentrations seems to be absent and is even slightly suggestive of an association counter to what was hypothesized.

**Table 2:** Association between exposure variables and type 2 diabetes prevalence: Odds Ratios with 95%-CI

Exposure Metric (Q:quartile)	Crude^a^	Adjusted^b^
**NO2-concentration **(µg·m^-3^)		

Q1: 8.8-14.2	*reference*	*Reference*

Q2: 14.2-15.2	0.98 (0.78-1.23)	1.03 (0.82-1.31)

Q3: 15.2-16.5	1.17 (0.94-1.45)	1.25 (0.99-1.56)

Q4: 16.5-36.0	0.80 (0.63-1.01)	0.80 (0.63-1.02)

**Distance to nearest main road **(m)		

Q1: 220-1610	*reference*	*reference*

Q2: 140-220	1.10 (0.87-1.39)	1.12 (0.88-1.42)

Q3: 74-140	1.22 (0.97-1.53)	1.17 (0.93-1.48)

Q4: 2-74	0.94 (0.74-1.19)	0.88 (0.70-1.13)

**Traffic flow at nearest main road **(veh·24 hrs^-1^)		

Q1: 5001-5871	*reference*	*reference*

Q2: 5871-7306	1.09 (0.87-1.39)	1.02 (0.81-1.29)

Q3: 7306-9670	0.98 (0.78-1.23)	1.03 (0.81-1.30)

Q4: 9670-35567	0.91 (0.72-1.16)	0.96 (0.75-1.22)

**Traffic in 250 m buffer **(10^3 ^veh·24 hrs^-1^)		

Q1: 63-516	*reference*	*reference*

Q2: 516-680	1.28 (1.01-1.61)	1.25 (0.99-1.59)

Q3: 680-882	1.15 (0.91-1.46)	1.13 (0.89-1.44)

Q4: 882-2007	1.13 (0.89-1.44)	1.09 (0.85-1.38)

^a^Crude model: not adjusted for any of the selected covariates.

^b^Adjusted model: adjusted for average monthly income, age (continuous) and gender.

The plots for distance to the nearest main road should be looked at reversely (highest distance means lowest exposure). To give a more true representation of the dispersion of air pollution from a road, the x-axis in the plots (distance) furthermore have a log scale. The plots, as well as the analyses per quartile, show an increasing prevalence with decreasing distance up until approximately the median. From there on, prevalence of diabetes drops and roughly at the 75-percentile, was below the prevalence at the largest distance (Table 2 and Figure 2). In some studies, distance to the nearest major road was dichotomized at cut-offs of 100 m or 250 m. In the present study, the age, gender and income adjusted OR for diabetes when living within 250 m of a main road was 1.09 (95%CI: 0.87-1.36) relative to those living further away. For living within 100 m this was 0.88 (0.74-1.05).

For traffic flow at the nearest main road, no association was seen with diabetes prevalence. Traffic in a 250 m buffer, however, suggested some (statistically non-significant) increased diabetes prevalence for the higher exposures (roughly the upper three quartiles) although again prevalence decreases among the highest exposed.

**Figure 3:**
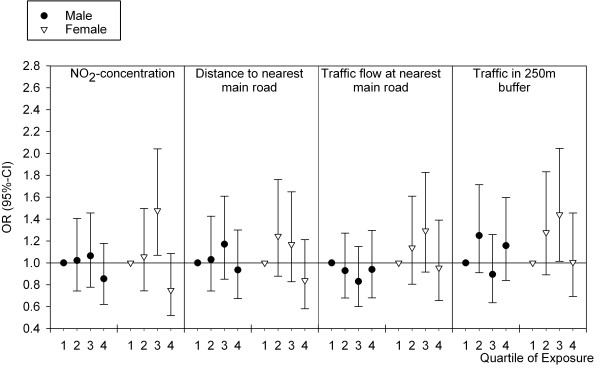
**Analyses stratified by gender**. Shown are ORs and 95%-CIs following from analyses adjusted for age and income.

Comparison of crude and adjusted models (Table 2 also Figure 2 vs. Additional File 1 Figure s1) demonstrates that inclusion of covariates in the adjusted models had little influence on the ORs and 95%-CIs. Additional adjustment for community did not change the results either (data not shown). Previous studies [2,3,5] suggest that gender could be an effect modifier, therefore analyses were stratified by gender (Figure 3). Patterns observed in the total population and described above seemed more pronounced among women than among men (also see Additional File 1 Figure s2). Statistically significantly increased odds were observed for modeled NO_2 _and traffic in a 250 m buffer (third quartile; 1.48 (1.07-2.04) and 1.44 (1.01-2.05), respectively). In regression analysis with exposure-gender interaction terms, however, the interaction was not statistically significant.

Sensitivity analyses were done to examine the potential confounding effect of BMI (Additional File 1 Table s2). In these analyses all participants with missing data on BMI (n = 98), all of which had previously diagnosed diabetes, were excluded. Crude and adjusted analyses showed slightly higher ORs and wider 95%-CIs than in the total population (Table 2). Additional adjustment for BMI did not affect exposure-response patterns to a great extent. We therefore concluded that BMI was not an important confounder for the association between traffic related air pollution and diabetes prevalence in this population. We furthermore tested for effect modification, regression analysis with exposure-BMI interaction terms, did not show statistically significant interaction.

**Figure 4:**
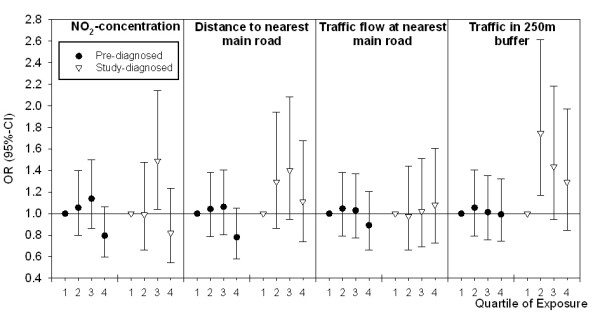
**Analyses stratified by type of diagnosis**. Shown are ORs and 95%-CIs following from analyses adjusted for age, gender and income. Dots are representing the ORs for self-reported previously doctor diagnosed diabetes (N = 7,805), triangles represent screening diagnosed diabetes (N = 7,612).

We also performed sensitivity analyses for the different types of diagnosis (self-reported previously doctor diagnosed vs. diagnosed by the extensive screening in this study, Figure 4), showing that the participants with screening diagnosed diabetes contribute importantly to the findings of this study.

## Discussion

In this study, smooth plots of exposure versus type 2 diabetes risk supported some association with traffic in a 250 m buffer. The prevalence of diabetes was (non-significantly) increased in the highest three quartiles compared to the lowest quartile, but did not increase with increasing quartile. Modeled NO_2_-concentration, distance to the nearest main road and traffic flow at the nearest main road were not associated with diabetes. Associations seemed to be stronger for women compared to men.

### Exposure in the study area

The area in which the Hoorn Screening Study was conducted has a relatively low level of air pollution, as documented with low NO_2_-concentrations, and small exposure contrasts. Doing studies in areas with low exposures and small contrasts has advantages and disadvantages. One important aspect of such studies is that knowledge of possible health effects of air pollution at concentrations below current standards could be gained. A disadvantage is the potentially low study power. The latter may have limited our ability to detect a consistent association with traffic-related air pollution. Since other studies [e.g. [22]] observed effects in areas with low exposure and limited contrast, and several studies have shown largely linear associations between air pollution and i.e. cardiopulmonary mortality [e.g. [23]], we considered exploration of a possible association in this study area to be worthwhile.

The limited ranges of exposure to traffic flow at the nearest main road and NO_2_-concentration could have contributed to inconsistent findings. For instance, the interquartile range for NO_2_-exposure in this study was only 2.3 μg/m^3^, while in previous studies on air pollution and type 2 diabetes [2,3] this ranged from 5.8 to 15.0 μg/m^3^. The relatively long tails at both ends of the exposure range, may furthermore have contributed to the absence of an exposure-response relation in this study: the range of exposure within the highest exposed quartile for NO_2 _(16.5-36.0 μg/m^3^) was much larger than the interquartile range. As shown in Figure 2, however, analysis exploiting the full contrast shows no increased odds with increased NO_2_-concentration either.

### Exposure-effect relation

In the present study, associations for different indicators of air pollution did not show consistent results. Whereas increased exposure as measured by traffic in a 250 m circular buffer was associated with slightly increased odds for type 2 diabetes, this pattern was less clear for distance to the nearest main road and modeled NO_2_-concentration and absent for traffic flow at the nearest main road. However, different associations for different exposure metrics were also observed in a cohort study on cardiovascular mortality in the Netherlands [17]. The exposure-response pattern for NO_2_-concentration and distance to the nearest main road in this study was similar, most likely due to the high correlation between the two variables. Distance to the nearest main road is a metric being increasingly used in policy practice, modeled NO_2_-concentration, however, is probably a more precise metric of exposure to traffic related air pollution.

### Potential misclassification of exposure

Exposure was characterized at the home-address. Despite high correlation between outdoor exposure at the home-address and overall exposure to traffic-related air pollution [19], personal differences in exposure, caused by, for instance, occupational or commuting exposure could have resulted in exposure misclassification. In addition, it is unknown for what time period participants had resided in the study area at the time of enrollment. Residential mobility among elderly persons in the Netherlands, however, tends to be low [24,25] and therefore we believe that estimated exposures in the present study represent long-term exposures of the study participants. Exposure and participant data were furthermore obtained at different moments in time. As the study area is a stable environment where no major modifications in housing or the road network have occurred in the past twenty years, we do not think that spatial variation of exposure has changed much over time. Recent studies showed reasonable long-term validity of land use regression models [26,27]. Indicators such as distance to the nearest main road may be even more stable over time than air pollution concentrations.

As exposure was characterized at the geocoded home-address, spatial error in the database that was used for geocoding may have contributed to exposure misclassification. Geocoding was done with ACN, of which the accuracy is high (93.5% located at centroid of the correct building, 6.0% at the centroid of the correct parcel [28]). We therefore believe that misclassification of exposure due to spatial error in the geo coded home-address, if any, is small.

### Study design

Ideally, epidemiological studies on the health effect of environmental exposures such as air pollution are conducted in a prospective cohort design. In order to study conditions such as type 2 diabetes in a cohort with sufficient power, a long follow-up time is needed and the size of the cohort has to be substantial. Since this is very time-consuming and costly, cross-sectional studies, such as the Hoorn Screening Study, can contribute to the understanding of such associations considerably in absence of cohort studies.

The Hoorn Screening Study is a cross-sectional study among a representative study population and the prevalence of diabetes is well-described. In questionnaire based studies, selection bias may be of importance. In the Hoorn Screening Study, selection bias was minimized by inviting all 50- to 75-year-old inhabitants of the study area to participate and non-response was low (20%) [12]. In general, type 2 diabetes remains undiagnosed in up to 30-55% of the cases. A strength of the present study is that many of these undiagnosed patients were detected [12]. About one third of the patients with type 2 diabetes in this study were diagnosed by the extensive screening procedure. Sensitivity analyses for type of diagnosis (self-reported vs. screen-detected, Figure 4) shows that the screening detected patients with type 2 diabetes contributed importantly to the findings of this study, a finding which may be of importance for setting up future studies. As subjects diagnosed in the screening were unaware of their disease, bias in especially this group seems unlikely. Although some misclassification might have occurred in the group of self-reported patients with type 2 diabetes, it is unlikely that this is related to exposure. This misclassification would therefore probably result in less pronounced effects, if any.

### Confounding and effect modification

Comparison of crude and adjusted models indicated little confounding of the relation between type 2 diabetes and exposure variables. We cannot rule out residual confounding by other unmeasured factors such as lifestyle, personal socio-economic status, etc. For example, no data were available on smoking status or prior cardiovascular disease, which are important risk factors for type 2 diabetes. In the three published epidemiological studies exploring the relation between traffic-related air pollution and diabetes, Brook et al. [2] adjusted for the same factors as in our study, whereas Krämer et al. [3] and Puett et al. [5] had more detailed individual information available. Neither of these studies however indicated those characteristics to be important confounders in the association between diabetes and air pollution. In several studies on cardiopulmonary health [29-31], it also seemed that adjustment for important risk factors such as smoking, had little influence on the relation between cardiopulmonary health and traffic-related air pollution. This is consistent with our findings, in which adjustment for gender, age and an indicator of socio-economic status (neighborhood average income) indicated that these were not confounders for the relation with traffic-related air pollution. Sensitivity analyses on the potential confounding effect of BMI showed furthermore no indication of confounding by BMI in this population (Additional File 1 Table s2, Model III vs. Model II) although residual confounding cannot completely be ruled out.

Krämer et al. [3] showed associations between traffic-related air pollution and incident type 2 diabetes among elderly women in a prospective study. For NO_2_, the adjusted relative risk (RR) was 1.42 (95%-CI: 1.16-1.73) per 19 μg/m^3^. Brook et al. [2] demonstrated a relation between modeled NO_2_-concentration and type 2 diabetes prevalence among women (OR 1.04 (1.00-1.08) per ppb), but not among men. Puett et al. [5] observed an increased hazard ratio of 1.14 (1.03-1.27) for living less than 50 m versus ≥200 m from a roadway among women. In our study, patterns observed in the full population seemed to be more pronounced among women, which is consistent with the studies by Brook, Puett and Krämer. In regression analysis, however, no statistically significant interaction by gender was shown. Among the potential explanations for a possible difference between men and women is accuracy of exposure estimation, which may be more accurate in women than in men. The women in this population are of a generation in which working outside of the home was rare. At the time of screening, women in this study therefore were more likely to have spent more time at home than men. Furthermore, susceptibility may differ between women and men.

## Conclusion

This study did not find consistent associations between type 2 diabetes prevalence and exposure to traffic related air pollution, though there were some indications for a relation with traffic in a 250 m buffer. Our study adds to the limited number of studies on air pollution as a risk factor for type 2 diabetes [2-5]. In contrast with previous epidemiological studies [2,3,5] we did not find consistent associations, though despite the limited level of exposure in the population studied, some indications for a relation were observed.

## List of abbreviations

95%-CI: 95% confidence interval; BMI: body mass index; GIS: geographical information system; NO_2_: nitrogen dioxide; OR: odds ratio; RR: relative risk.

## Competing interests

The authors declare that they have no competing interests.

## Authors' contributions

MD, SM, UG, JD and BB substantially contributed to conception and design of the study, acquisition, analysis and interpretation of data; drafted and revised the article and approved the final version. KvdH, MA, RvS, GH substantially contributed to design and interpretation of data, revised the article critically and approved the final version. PF, GN, CS substantially contributed to acquisition of data, revised the article and approved of the final version.

## Supplementary Material

Additional file 1 Table s1 **Supplemental Material dijkema diabetes**. Click here for file
