# Supplementary material for: Long-term Exposure to Traffic-related Air Pollution and Type 2 Diabetes Prevalence in a Cross-sectional Screening-study in the Netherlands
Source: Environ Health. 2011 Sep 5;10:76. doi: 10.1186/1476-069X-10-76 (PMC3200985; doi:10.1186/1476-069X-10-76)
Supplement: Additional file 1 Table s1 — Supplemental Material dijkema diabetes. [file 1476-069X-10-76-S1.DOC]

**SUPPLEMENTAL MATERIAL to:**

**Long-term Exposure to Traffic-related Air Pollution and Type 2 Diabetes Prevalence in the Netherlands**

M Dijkema, S Mallant, U Gehring, K van den Hurk, M Alssema,R van Strien, P Fischer, G Nijpels, C Stehouwer, G Hoek, J Dekker,B Brunekreef

**Index:**

[Table s1: 2](#__RefHeading___Toc302460867)

[Figure s1: 3](#__RefHeading___Toc302460868)

[Figure s2: 4](#__RefHeading___Toc302460869)

[Table s2: 5](#__RefHeading___Toc302460870)

Table s1: Distribution of exposure variables (and the predictors of the NO2 LUR model)

for the total population and for the people with and without Type 2 Diabetes separately.

| **Exposure variable** | **minimum** | **10th percentile** | **25th percentile** | **median** | **75th percentile** | **90th percentile** | **maximum** |
| --- | --- | --- | --- | --- | --- | --- | --- |
| **TOTAL POPULATION** |  |  |  |  |  |  |  |
| Modeled NO­2-concentration  (µgm-3) | 8.8 | 13.5 | 14.2 | 15.2 | 16.5 | 18.4 | 36.0 |
| **Predictors:** |  |  |  |  |  |  |  |
| *Background concentration (µgm-3)* | *13.4* | *13.5* | *13.5* | *13.5* | *13.6* | *14.6* | *16.8* |
| *Traffic flow at nearest road (veh24hrs-1)* | *1,225* | *1,225* | *1,225* | *1,225* | *1,225* | *4,745* | *35,567* |
| *Distance to nearest main road (m)* | *2* | *30* | *74* | *140* | *220* | *338* | *1,610* |
| *Residential land use in 5km buffer (%)* | *4* | *9* | *10* | *13* | *14* | *15* | *17* |
| Distance nearest main road  (m) | 2 | 30 | 74 | 140 | 220 | 338 | 1610 |
| Traffic flow nearest main road  (veh24hrs-1) | 5,001 | 5,115 | 5,871 | 7,306 | 9,670 | 14,970 | 35,567 |
| Traffic within 250 m buffer  (10324hrs-1) | 63 | 367 | 516 | 680 | 882 | 1,158 | 2,007 |
| **TYPE 2 DIABETES** |  |  |  |  |  |  |  |
| Modeled NO­2-concentration  (µgm-3) | 11.2 | 13.5 | 14.3 | 15.2 | 16.3 | 18.0 | 26.4 |
| **Predictors:** |  |  |  |  |  |  |  |
| *Background concentration (µgm-3)* | *13.4* | *13.5* | *13.5* | *13.5* | *13.6* | *13.7* | *16.8* |
| *Traffic flow at nearest road (veh24hrs-1)* | *1,225* | *1,225* | *1,225* | *1,225* | *1,225* | *1,804* | *17,304* |
| *Distance to nearest main road (m)* | *4* | *35* | *81* | *138* | *208* | *324* | *1,266* |
| *Residential land use in 5km buffer (%)* | *8* | *9* | *10* | *13* | *14* | *15* | *16* |
| Distance nearest main road  (m) | 4 | 35 | 81 | 138 | 208 | 324 | 1,266 |
| Traffic flow nearest main road  (veh24hrs-1) | 5,001 | 5,115 | 5,871 | 6,790 | 9,278 | 14,736 | 35,567 |
| Traffic within 250 m buffer  (10324hrs-1) | 65 | 392 | 531 | 683 | 878 | 1,146 | 1,860 |
| **NO TYPE 2 DIABETES** |  |  |  |  |  |  |  |
| Modeled NO­2-concentration  (µgm-3) | 8.8 | 13.5 | 14.3 | 15.2 | 16.6 | 18.4 | 36.0 |
| **Predictors:** |  |  |  |  |  |  |  |
| *Background concentration (µgm-3)* | *13.4* | *13.5* | *13.5* | *13.5* | *13.6* | *14.6* | *16.8* |
| *Traffic flow at nearest road (veh24hrs-1)* | *1,225* | *1,225* | *1,225* | *1,225* | *1,225* | *4,745* | *35,567* |
| *Distance to nearest main road (m)* | *2* | *30* | *73* | *140* | *220* | *343* | *1,610* |
| *Residential land use in 5km buffer (%)* | *4* | *9* | *10* | *13* | *14* | *15* | *17* |
| Distance nearest main road  (m) | 2 | 30 | 73 | 140 | 220 | 343 | 1,610 |
| Traffic flow nearest main road  (veh24hrs-1) | 5,001 | 5,115 | 5,871 | 7,306 | 9,670 | 14,970 | 35,567 |
| Traffic within 250 m buffer  (10324hrs-1) | 63 | 367 | 516 | 680 | 882 | 1,158 | 2,006 |

Figure s1: Smooth crude associations (OR and 95%-CI) between exposure variables and type 2 diabetes prevalence. Box plots on the x-axis present distribution of exposure variables.


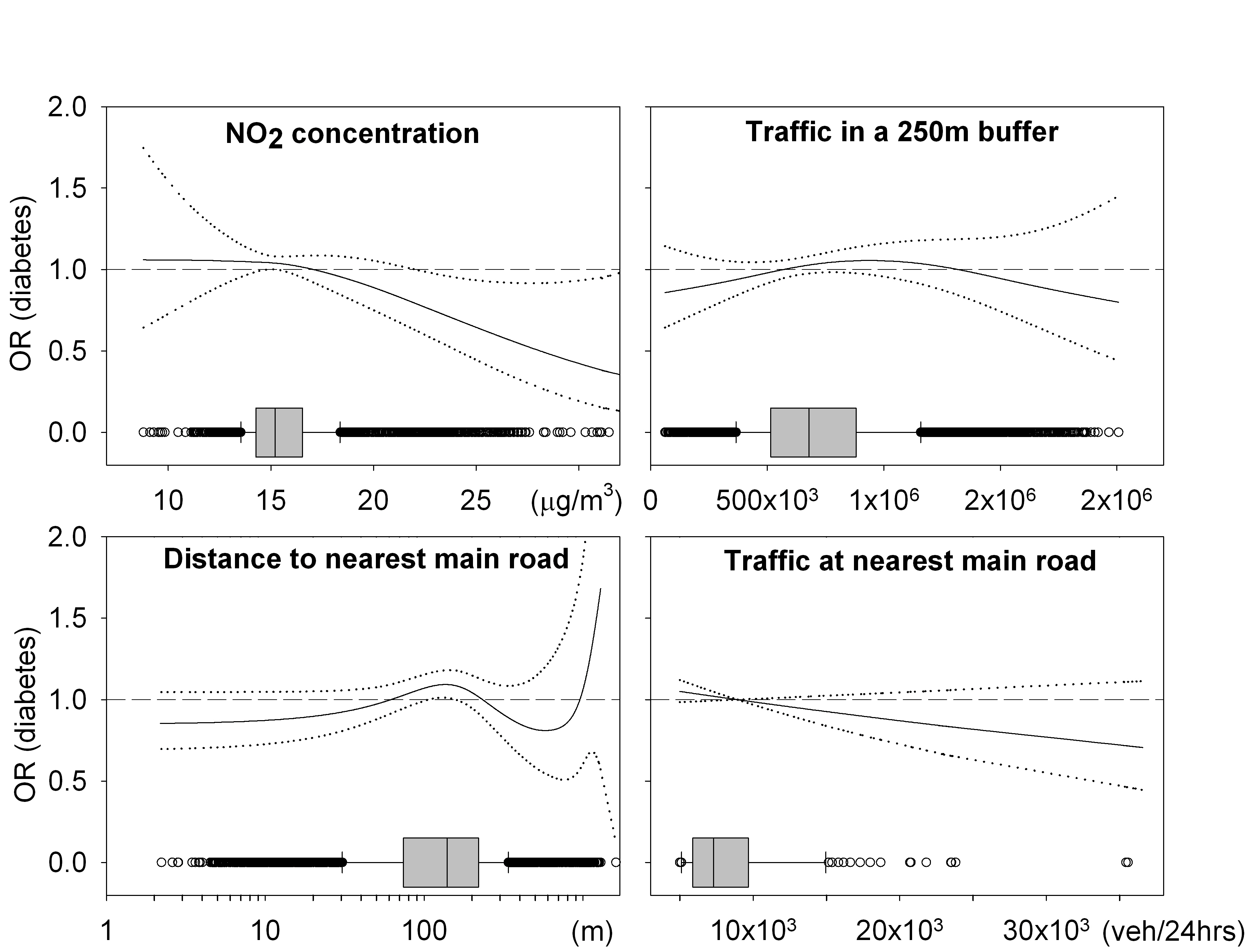


Figure s2: Smooth (age and income) adjusted associations (OR and 95%-CI) between exposure variables and type 2 diabetes prevalence, stratified by gender. Lines on the x-axis represent the distribution of exposure.


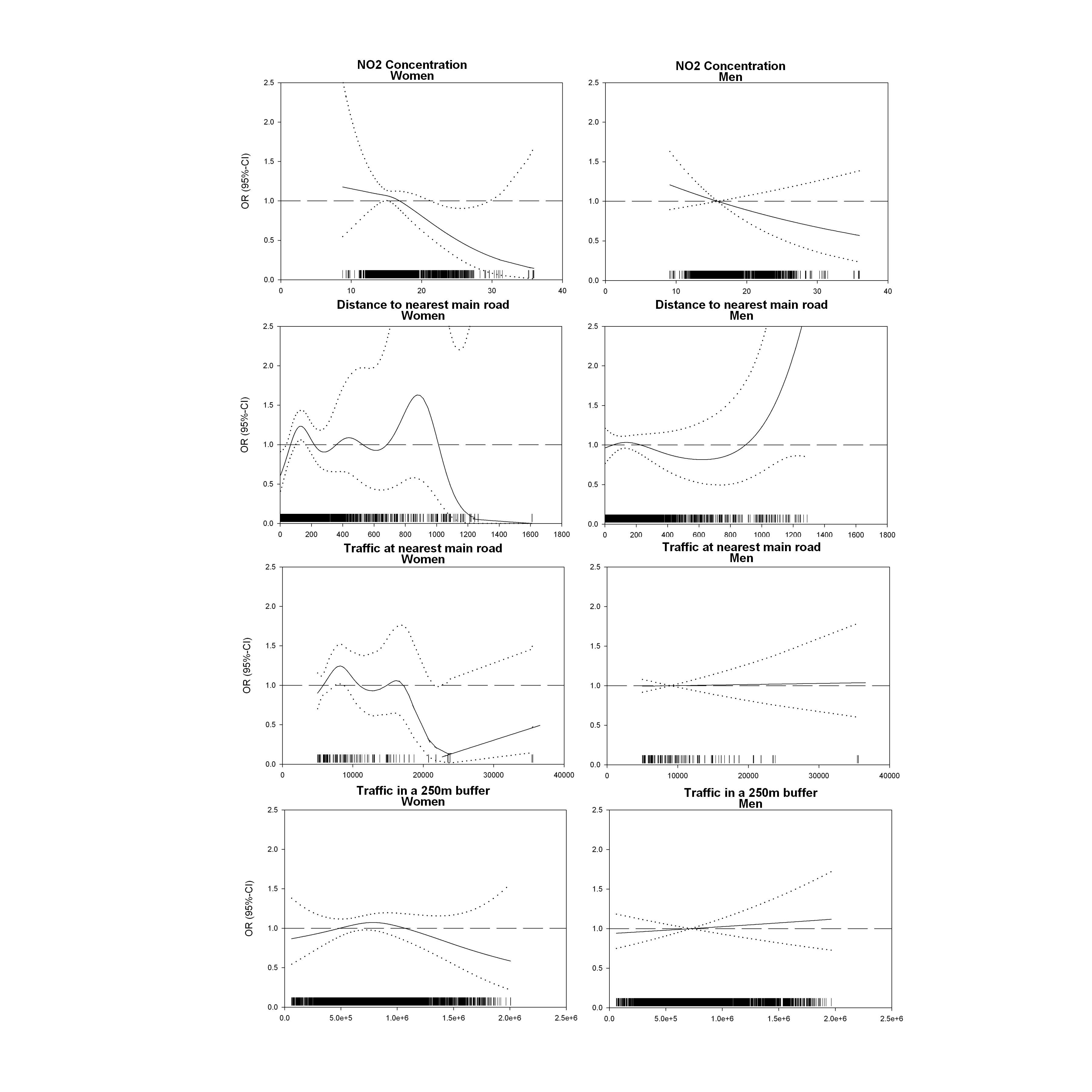


Table s2: Association between exposure variables and type 2 diabetes prevalence:

sensitivity analyses for BMI within a population of 7,920 participants, including 521 (7%) participants with type 2 diabetes. Excluded are 98 participants (1% of total population) with previously diagnosed type 2 diabetes, because of missing BMI data.

|  | Model I: Crudea | Model II: Adjustedb | Model III: Adjusted + BMIc |
| --- | --- | --- | --- |
| **NO2-concentration** | | | |
| Q1 | *reference* | *reference* | *reference* |
| Q2 | 1.08 (0.84-1.39) | 1.14 (0.88-1.47) | 1.11 (0.85-1.44) |
| Q3 | 1.31 (1.03-1.66) | 1.39 (1.09-1.78) | 1.39 (1.08-1.78) |
| Q4 | 0.84 (0.65-1.09) | 0.84 (0.64-1.09) | 0.84 (0.64-1.10) |
| **Distance to nearest main road** | | | |
| Q1 | *reference* | *reference* | *reference* |
| Q2 | 1.18 (0.92-1.54) | 1.21 (0.93-1.57) | 1.20 (0.92-1.56) |
| Q3 | 1.36 (1.06-1.74) | 1.30 (1.00-1.67) | 1.30 (0.99-1.68) |
| Q4 | 1.05 (0.80-1.36) | 0.98 (0.75-1.28) | 0.99 (0.75-1.30) |
| **Traffic flow at nearest main road** | | | |
| Q1 | *reference* | *reference* | *reference* |
| Q2 | 1.08 (0.84-1.38) | 1.01 (0.78-1.29) | 1.02 (0.79-1.32) |
| Q3 | 0.96 (0.75-1.23) | 0.99 (0.78-1.28) | 1.01 (0.78-1.31) |
| Q4 | 0.89 (0.69-1.15) | 0.93 (0.71-1.21) | 0.91 (0.70-1.19) |
| **Traffic in 250m buffer** | | | |
| Q1 | *reference* | *reference* | *reference* |
| Q2 | 1.43 (1.10-1.84) | 1.40 (1.08-1.81) | 1.34 (1.03-1.75) |
| Q3 | 1.25 (0.96-1.62) | 1.22 (0.94-1.59) | 1.20 (0.92-1.57) |
| Q4 | 1.24 (0.96-1.62) | 1.19 (0.91-1.55) | 1.19 (0.91-1.56) |

aModel I (crude model): crude relation between type 2 diabetes and exposure variables; not adjusted for any of the a priori selected covariates.

bModel II (adjusted model): adjusted for a priori selected covariates average monthly income, age (continuous) and gender.

cModel III (adjusted model +BMI): adjusted for a priori selected covariates and BMI.
